# A Report on New Remedies

**Published:** 1864-09

**Authors:** Edward B. Stevens

**Affiliations:** Editor Cincinnati Lancet & Observer


### A REPORT ON NEW REMEDIES.

[Read before the Ohio State Medical Society, White Sulphur Springs, June 21, 22, 1864.]

By Edward B. Stevens, M. D., Editor Cincinnati Lancet & Observer.

The present report of your committee on new remedies will be mainly a continuation of the report of last year, and therefore will be devoted to the notice of such remedies aud preparations as have recently been presented for favorable consideration; nevertheless some general remarks in connection will not be entirely out of place.

In an editorial in the *Buffalo Medical Journal*, by Dr. Miner, we find some remarks on Drugs and their use in the treatment of disease that are corrollary to views expressed in a former report of your committee, showing especially that the tendency of scientific medicine in its steady progress is toward the treatment of disease with less drug medication—their proper use being rather for the assistance of the vital force in its instinctive resistance of pathological changes, and their conduct to safe terminations. We quote a brief paragraph or two expressive of these ideas:

"Disease is almost everywhere over-treated, and nothing can be more plain or more easily demonstrated than this proposition. If we did not know it was true we should be glad to speak otherwise. It did appear at one time that the vagaries of Hahnneman were to be adopted in a degree to prevent somewhat the injurious abuse of medicine, but even the belief that Homœpathic remedies would at least do no harm has long since been dissipated with the knowledge that even the disciples of this monstrous delusion are drugged more extensively and more dangerously than any other ; they are literally fed on drugs, in doses which would do honor to the chivalrous days of 'heroic practice.'

"The progress of true medical science has greatly qualified our estimate of the value of mere medicine in the treatment of disease. The sore that used to be treated with an unguent composed of twenty ingredients, heals under moist lint when placed in proper position, or supported by the stimulus of gentle pressure. The pneumonia that used to be attacked with heroic remedies—bleeding, antimony and calomel—now gets well with horizontal position and small doses of Dovers' powder. Inflammation even of the serous membranes, which formerly received most active medication is now observed to terminate favorably, if pain is abated and sleep obtained by a full anodyne. The more painless or even pleasent a physician can make his treatment, the more he can divest it of irritating and disturbing characters, the better is it, and the greater and more acceptable is he. The chief characteristic of advancing therapeutics, is to watch the natural course of disease, to regard pathological processes only as modifications of physiological ones, with a natural tendency to terminate in harmonious and healthy action when the obstacles are overcome which the pathological processes themselves were put in action to remove. We often see in the worst forms of disease 'an effort of nature to throw off the morbific matter, and thus cure the patient.' All this is done without any detraction from the dignity and importance of the physician; he is indeed much more worthy of public admiration and confidence than he who would attain the same result by the most active medical warfare.

"Physicians never talked so modestly about 'curing' disease as now, and those who excel in this modesty do most towards the furtherance of the object."

*U. S. Pharmacopiœa.*—During the past year the regular decennial revision of the United States Pharmacopiœa has been issued, by authority of National Convention for revising the Pharmacopiœa held in the City of Washington in the year 1860. It is of course to be expected that with the completion of each decennial interval, the progress of medical science will bring with it many modifications in the opinion of practitioners as to the value of remedial agents; and this last revision while it is another evidence that our science has not reached a state of perfection—is also an evidence of the steady and pains-taking progress we are making in this department of our profession.

A Pharmacopiœa of the United States is not merely a series of formulæ for the best mode of presenting uniform preparations—but it is for the time a declaration of what the experience of the profession has decided shall be considered its standard officinal preparations.

As some evidence of the change which experience has brought in the views of practitioners—we remark that in the list of the *Materia Medica* proper—twenty-six articles have been dismissed as useless or so inferior as to be unworthy of a regular place as officinal articles; and from the list of officinal preparations—twenty-seven have been in like manner dismissed: on the other hand, fifty-five articles have been considered of sufficient value to be added to the *Materia Medica*—and one hundred and eleven preparations. These are not however to be strictly regarded as *new remedies*—they are the new remedies which the experience of ten years has approved—and a large proportion are already in general use before the mere declaration of the Pharmacopiœa had made them officinal.

Of course we shall have next in order—and at an early date—a fresh edition of the United States Dispensatory conforming to this modified Pharmacopiœa, which will be looked for with interest by the profession.

In this connection it is but fitting that we acknowledge the eminent services of that diligent "hand maid" *Pharmacy.* In this country the plodding compounder of drugs has within comparatively a recent period, risen rapidly to the dignity of an independent and worthy profession; in this we should sincerely rejoice—for the elevation of Pharmacy represents in an important degree the progress of medical science. While we should systematically frown upon that villainous nondescript which continues in a large degree to infest our cities and larger villages—who is half druggist, half doctor; bleeds, pulls teeth and treats gonorrhœa; who fawns on the members of the profession for their prescription patronage, and sneers at them to their patients; who resorts to all scurvy tricks for a consideration; nevertheless we say all honor to the true Pharmaceutist—let us strive to draw the distinction in our esteem—and so far as may be draw the distinction in our patronage.

During the past year or two, the *London Lancet* has published a series of articles on what the contributor is pleased to style *new remedies.* Some carefulness in the reading of these articles incline us to the opinion that a large amount of trash has been gathered up, with a few really valuable contributions; quite a number of remedies are taken at second hand from the representations of Eclectic and Botanic practitioners of this country, who are by no means regarded with a prophet's honor at home. Others are treated of as new which have been long known in this country and used by all classes of practitioners—as for example the *Phitolacca decandra,* (poke root.) The several individual articles that we propose to notice are too disconnected to suggest any systematic order, we therefore take up first—

*Substitutes for Quinia; Cinchonine.*—In the series of articles we allude to, we have *cinchonine* presented as a reliable anti-periodic, in all respects equally efficacious as a remedy, though in doses one-third larger than quinine. The cinchonine has the advantage of being less bitter in taste, and much cheaper. The cinchonine is especially commended in a communication from Dr. McPherson, who has had a lengthy and extended opportunity for observation in the fevers of the East Indies. Inasmuch as the sources of supply of quinine are becoming every year more limited, it becomes a very important inquiry to test a reliable substitute—or even a substitute which shall be reliable for many purposes. It is stated that the supply of cinchonia is both cheap and abundant. The *Swamp Ash* or *Fraxinus Nitgra,* is offered as another substitute for the sulphate of quinine. In an article in the *Cincinnati Lancet and Observer* for April, 1864, Dr. Denny of Albion, Indiana, reports an experience of ten years in the use of the swamp ash. He administers the remedy in the form of a syruppy decoction or fluid extract of the inner bark, in doses of a table spoonful, frequently repeated during the state of apyrexia, adding a full dose of opium to the last dose of swamp ash in anticipation of the expected paroxysm. He says "ever since 1854 all my cases of intermittents have been thus treated, and I candidly aver has never failed to arrest the disease." He further expresses the opinion, from his observation that relapses of ague thus treated are less apt to return.

*Phloridzine,* is another remedy which has some claims to professional regard, as in some degree a substitute for quinia. As long ago as the year 1856, some favorable notices of phloridzine appeared in the medical journals. We find in the *Medical Observer* of Cincinnati some account of its use by physicians of that city in the treatment of intermittent diseases. Dr. De Ricci revives attention to this remedy in a recent article in the *Dublin Quarterly Journal of Medical Science,* August, 1864, as follows:

"*Phloridzine* is a neutral principle existing in considerable quantities in the bark of the root of the apple, plum, and cherry trees, but principally in the apple tree. It appears in the market in the form of a dirty whitish powder, consisting of broken up silky needles, somewhat resembling quinine which has not been well bleached, and when rubbed between the fingers it has a soft velvety feel, very like that of French chalk. When chrystalized by slow cooling from a dilute solution, previously treated with fresh prepared animal charcoal, phloridzine may be obtained perfectly white, and in the form of long silk needles. Its taste is peculiar, being bitter at first, but afterwards somewhat sweetish, with a flavor of apples. Phloridzine differs from quinine by containing no nitrogen in its chemical composition, but in this respect it resembles salacine, to which it is much allied. Like salacine it does not combine with acids to form salts, is very soluble in alcohol, ether, or boiling water, but requires one thousand parts of cold water for solution.

[CONCLUDED IN NEXT NUMBER.]

